# Sequential everolimus for angiomyolipoma associated with tuberous sclerosis complex: a prospective cohort study

**DOI:** 10.1186/s13023-021-01913-2

**Published:** 2021-06-14

**Authors:** Liangyou Gu, Cheng Peng, Fan Zhang, Cunjin Fang, Gang Guo

**Affiliations:** 1 Department of Urology, the Third Medical Centre, Chinese PLA General Hospital, 69 Yong Ding Road, Beijing, 100039 China; 2 School of Pharmacy, Capital Medical University, Beijing, China

**Keywords:** Angiomyolipoma, Tuberous sclerosis complex, Mutation, Everolimus, Mammalian target of rapamyoin

## Abstract

**Background:**

To evaluate the efficacy, safety and health economics of sequential everolimus in treating angiomyolipoma (AML) associated with tuberous sclerosis complex (TSC).

**Methods:**

In this prospective cohort study, patients met the inclusion criteria received standard or sequential treatment according to their willingness. All patients received an initial dose of everolimus (10 mg oral, once a day) for 3 months. The standard treatment group maintained 10 mg QD for 12 months, while the sequential treatment group reduced the dose to 5 mg QD from the 4th month. The efficacy, serum everolimus concentration and safety were evaluated at 1, 3, 6, 9 and 12 months after treatment. The primary efficacy endpoint was the proportion of patients with confirmed angiomyolipoma response of at least a 50% reduction in the total volume of target AML relative to baseline.

**Results:**

Between June 1, 2016 and June 1, 2017, a total of 53 patients were included. Twenty-three patients received standard treatment, 30 patients received sequential treatment. At 1, 3, 6, 9 and 12 months after treatment, the proportion of patients whose total target tumor volume decreased by ≥ 50% from baseline was 39.1% versus 36.7%, 43.5% versus 56.7%, 47.8% versus 50%, 47.8% versus 60% and 47.8% versus 23.3% respectively (*P* > 0.05 for all). The overall response rate of skin lesions in the two groups was 40.4%, and the response rates of skin lesions at different times were similar for two groups (*P* > 0.05 for all). Major adverse effects (AEs) included mouth ulceration, hypertriglyceridemia, hypercholesterolemia, menstrual disorders. There was no significant difference between the two groups in the incidence of AEs at 3 months after treatment. The incidence of overall and grade 3/4 AEs at 12 months after treatment were significantly lower in the sequential treatment group. The average direct cost of the two groups in 12 months was $15,466 and $11,120, respectively.

**Conclusions:**

Compared to standard treatment, sequential treatment was equally effective, with a lower incidence of adverse events and a lower direct cost, suggesting that it may be an alternative treatment for AML associated with TSC.

## Introduction

Angiomyolipoma (AML), a common benign tumor of kidney, can be sporadic or the renal manifestation of tuberous sclerosis complex (TSC). TSC is an autosomal dominant syndrome involving multiple organs originating from three embryonic layers. It is mainly caused by inactivation mutations of TSC1 and/or TSC2 genes, and the lesion progresses gradually with age [1]. Compared with sporadic renal AML, TSC-related renal AML is mostly bilateral, prone to severe consequences such as bleeding and renal failure, and is the main cause of death in adult TSC patients [2]. In recent years, everolimus and other mammalian target of rapamyoin (mTOR) inhibitors have been used in the treatment of TSC-related AML and achieved satisfactory results, which have been recommended as first-line treatment by guidelines [3]. However, whether the 10 mg daily regimen recommended by previous studies is also applicable to Chinese patients remains controversial. Hence, to explore appropriate administration regimens for TSC-AML patients in China, we conducted a prospective cohort study of individualized everolimus therapy in TSC-associated renal AML patients.

## Methods

### Patients

All patients were ≥ 18 years old, each patient had at least one renal angiomyolipoma (RAML) with a diameter of at least 3 cm, and was clearly diagnosed as tuberous sclerosis complex (TSC)-related renal AML by clinical or genetic diagnostic criteria. The diagnostic criteria were the 2012 version of the clinical diagnostic criteria for TSC [4]. Gene diagnosis is based on the detection of any pathogenic mutation of the TSC1 or TSC2 genes by high-throughput second-generation sequencing technology. Patients were excluded if their AML required surgery or they had AML related bleeding or embolization in 6 months prior to study inclusion.

From June 2016 to June 2017, a total of 53 patients with confirmed TSC-RAML met the inclusion criteria and were included in the study. This study was approved by the medical Ethics Committee of our hospital, and the informed consent of each patient was obtained. The present study was registered on Chinese Clinical Trial Registry (ChiCTR-OPN-16008236).

### Study design

It was a prospective cohort study performed in our single center. For each patient met the inclusion criteria, we have detailed the purpose and design of our study. Furthermore, for standard and sequential treatment, the detailed strategy, potential advantages and disadvantages (tumor control, adverse effect, direct cost) were emphatically explained to each patient. After obtaining the informed consent, according to their willingness, patients were allocated to two groups. All patients received an initial dose of everolimus (10 mg oral, once a day) for 3 months. The standard treatment group maintained 10 mg QD for 12 months, while the sequential treatment group reduced the dose to 5 mg QD from the 4th month.

### Assessment and outcomes

The follow-up period was 12 months, and the efficacy, blood drug concentration and safety were evaluated at 1, 3, 6, 9 and 12 months after treatment. Efficacy evaluation was performed by magnetic resonance imaging (Siemens Magnetom Skyra 3.0 T), tumor volume was estimated using the three-dimensional imaging software. CTCAE V4.0 standard was used for safety evaluation. Fasting serum of the patients before taking the drug in the morning was tested for serum trough concentration of rapamycin (x) with the immunological method, and then the serum trough concentration of everolimus (y) was obtained by formula conversion, the specific formula is y = 1.29x − 0.068 [5]. The primary study endpoint was a reduction of RAML volume by 50% or more relative to baseline. Main observed indicators included proportion of patients with ≥ 50% reduction in RAML volume relative to baseline, blood concentration of everolimus, incidence of adverse reactions, reaction rate of cutaneous lesions, and indicators of health economics. In case of uncontrolled grade 3/4 adverse drug reactions, tumor progression, or renal tumor rupture and hemorrhage, the patient should reduce the drug dose or terminate the treatment in time. Blood routine examination, urine routine examination, blood biochemistry, rapamycin blood concentration, chest X-ray, renal MRI and a digital photo of skin lesions were performed at 1, 3, 6, 9 and 12 months after treatment.

### Statistical analysis

For continuous variables, the normality has been assessed by the Kolmogorov–Smirnov test. Since all continuous variables were non-normal distributed, they were described as median and interquartile range. The Wilcoxon rank sum test was used to test the differences for continuous variables. The Pearson chi-squared or Fishers’ exact test was applied to test the differences for categorical variables. All statistical analyses were performed using R software (version 3.3.1). Bilateral *P* < 0.05 was considered statistically significant.

## Results

Between June 1, 2016 and June 1, 2017, a total of 53 patients were included in this study. Twenty-three patients received standard treatment, 30 patients received sequential treatment. All patients completed 1 year of drug therapy and follow-up. There were no cases of tumor rupture, bleeding or nephrectomy during treatment and follow-up, and no disease progression occurred for all patients during follow-up.

Baseline features and disease characteristics were comparable between the two groups (Table 1). Overall, the median age was 28 (24–35) years for all patients, 53 (100%) patients have bilateral AML, 35 (66%) patients had an AML with the longest diameter of at least 8 cm. TSC2 mutation occurred in 34 (64%) patients. Twenty-five (47%) patients experienced previous treatment, including radical nephrectomy for 6 patients, partial nephrectomy for 7 patients, artery embolization for 12 patients.

**Table 1:** Baseline patient demographic and disease characteristics

Variables	All	Standard	Sequential	*P* value
No. patients	53	23	30	
Age, years, median (IQR)	28 (24–35)	32 (26–36)	28 (24–32)	0.815
Gender, n (%)				0.877
Male	11 (21)	5 (22)	6 (20)	
Female	42 (79)	18 (78)	24 (80)	
Clinical diagnosis of TSC, n (%)				
With LAM	31 (58)	13 (57)	18 (60)	0.799
With SEGA	28 (53)	12 (52)	16 (53)	0.933
≥ 1 skin lesion	48 (91)	21 (91)	27 (90)	0.872
History of epilepsy	17 (32)	7 (30)	10 (33)	0.823
Family history	11 (21)	5 (22)	6 (20)	0.877
TSC1/2, n (%)				1.000
TSC1 mutation	0 (0)	0 (0)	0 (0)	
TSC2 mutation	34 (64)	15 (65)	19 (63)	
No mutation	19 (36)	8 (35)	11 (37)	
Previous treatment, n (%)				
Radical nephrectomy	6 (11)	2 (9)	4 (13)	0.597
Partial nephrectomy	7 (13)	3 (13)	4 (13)	0.975
Artery embolization	12 (23)	5 (22)	7 (23)	0.891
Longest diameter of largest AML, n (%)				0.912
≥ 8 cm	35 (66)	15 (65)	20 (67)	
≥ 3 cm, < 8 cm	18 (34)	8 (35)	10 (33)	
Bilateral AML, n (%)	53 (100)	23 (100)	30 (100)	1.000
Measurable number of AML, n (%)				0.912
1–5	18 (34)	8 (35)	10 (33)	
6–10	35 (66)	15 (65)	20 (67)	
Total volume of AML, cm^3^, median (IQR)	840 (275–1450)	500 (220–1500)	880 (300–1425)	0.815

IQR, interquartile range; TSC, tuberous sclerosis complex; LAM, lymphangioleiomyomatosis; SEGA: subependymal giant cell astrocytoma; AML, angiomyolipoma

**Fig. 1:**
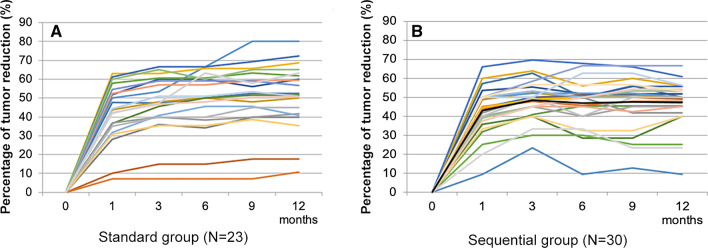
Rate of tumor volume reduction in both groups. **A** Standard group; **B** Sequential group

**Fig. 2:**
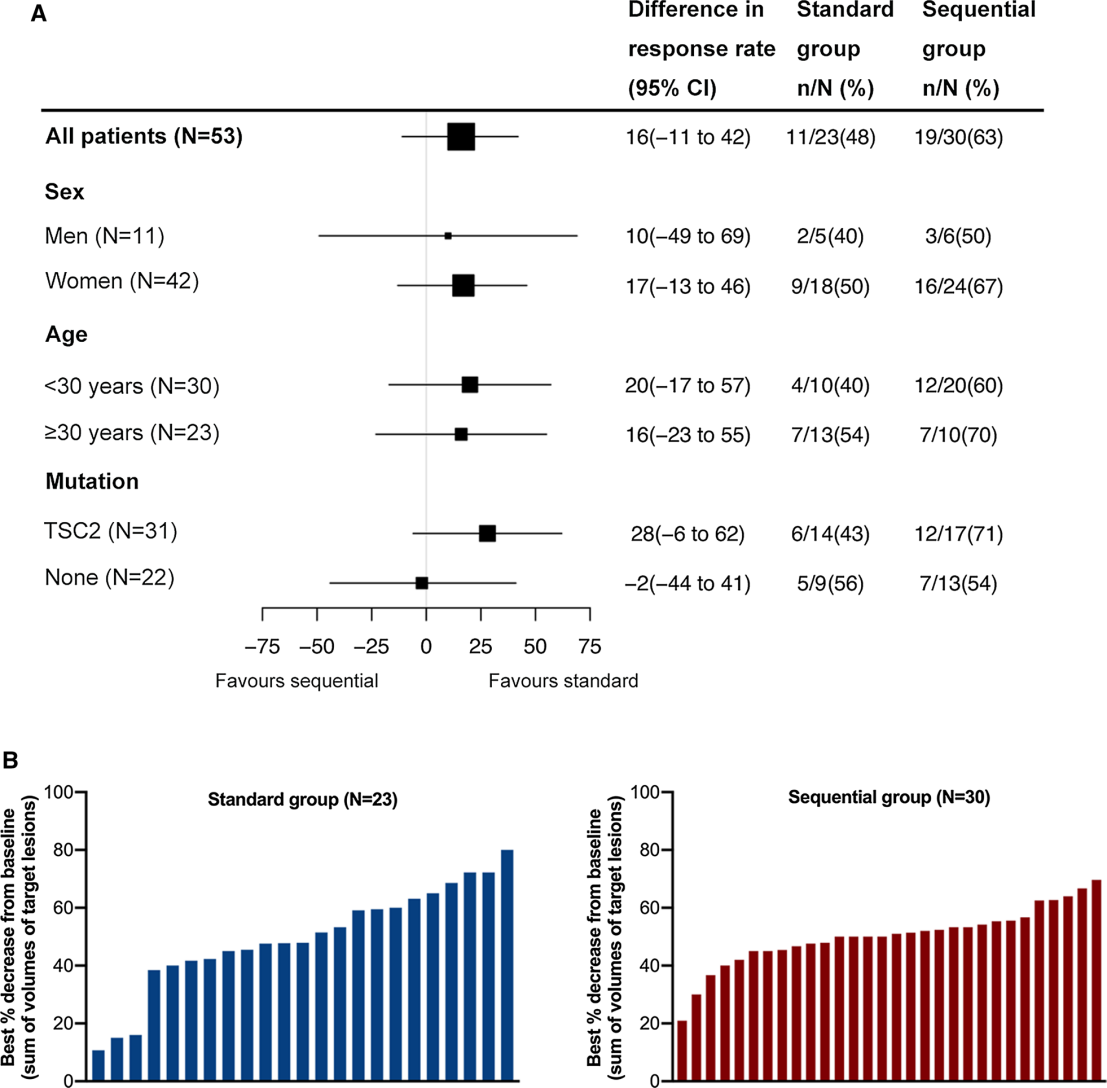
Magnitude of treatment effect. **A** Tumor response rates by subgroups (sex, age, mutation); **B** Best percentage change from baseline in the sum of volumes of target lesions in both groups

Most of the patients showed rapid tumor volume reduction at the first month of drug treatment. The average tumor response time was 1.2 months, and the tumor volume changes tended to be stable after 3 months of treatment (Fig. 1). At 1, 3, 6, 9 and 12 months after treatment, the proportion of patients whose total target tumor volume decreased by ≥ 50% from baseline was 39.1% versus 36.7%, 43.5% versus 56.7%, 47.8% versus 50%, 47.8% versus 60% and 47.8% versus 23.3% for standard and sequential treatment group respectively, with no statistically significant difference between the two groups (*P* > 0.05 for all). Subgroup analyses according to age, sex and mutation type did not identify significant differences in treatment effect between the two groups (Fig. 2). The overall response rate of skin lesions in the two groups was 40.4%, and the response rates of skin lesions at different times were 26.3% versus 25.0%, 36.8% versus 35.7%, 42.1% versus 39.3%, 42.1% versus 39.3%, and 42.1% versus 39.3% for standard and sequential treatment group respectively, with no significant statistical difference between the two groups (*P* > 0.05 for all).

Major adverse reactions during treatment included mouth ulceration, rashes, urinary tract infection, menstrual disorders, anemia, liver function abnormalities, noninfectious pneumonia. Of them, mouth ulceration occurred in 98.1% patients, hypertriglyceridemia occurred in 56.6% patients, hypercholesterolemia occurred in 58.5% patients, menstrual disorder occurred in 34.0% patients, urinary tract infection occurred in 30.2% patients. Most of them were grade 1–2, and the cumulative incidence of grade 3/4 adverse reactions was 27.7%. There was no significant difference between the two groups in the incidence of total (*P* = 0.780) and grade 3–4 (*P* = 0.690) adverse reactions after the 3 months treatment (Table 2). The incidence of total (*P* = 0.010) and grade 3/4 (*P* = 0.008) adverse reactions in the sequential treatment group after 12 months were significantly lower than those in the standard treatment group (Table 3). For the first 3 months and latter 9 months, the incidence of drug reduction or interruption was 68.4% versus 71.4% and 57.9% versus 17.9%, respectively. At 3 months and 6 months of administration, the mean serum trough concentration of everolimus for the two groups were 19.63 ng/ml versus 20.09 ng/ml and 15.45 ng/ml versus 9.07 ng/ml, respectively. The cumulative average treatment cost of the two groups in 12 months was $15,466 and $11,120, respectively, showing a statistically significant difference.

**Table 2:** Main adverse events of any cause in 3 months

Variables	Standard (n = 23)		Sequential (n = 30)		*P* value
	All grade	Grade 3/4	All Grade	Grade 3/4	
Total	23 (100)	6 (26)	29 (97)	9 (30)	0.780/0.690
Mouth ulceration	23 (100)	3 (13)	29 (97)	4 (13)	
Hypertriglyceridemia	14 (61)	2 (9)	16 (53)	2 (7)	
Hypercholesterolemia	14 (61)	2 (9)	17 (57)	2 (7)	
Menstrual disorder	8 (44)	1 (4)	10 (42)	1 (4)	
Urinary tract infection	8 (35)	0 (0)	8 (27)	1 (4)	
Rash	5 (22)	1 (4)	7 (23)	1 (4)	
Headache	5 (22)	1 (4)	6 (20)	0 (0)	
Gastritis	5 (22)	0 (0)	6 (20)	1 (4)	
Liver dysfunction	4 (17)	2 (9)	4 (13)	2 (7)	
Fatigue	4 (17)	1 (4)	6 (20)	1 (4)	
Non-infectious pneumonia	4 (17)	0 (0)	3 (10)	0 (0)	
Anemia	3 (13)	0 (0)	5 (17)	1 (4)	
Renal dysfunction	3 (13)	0 (0)	4 (13)	0 (0)	
Epileptic seizure	2 (9)	0 (0)	1 (4)	0 (0)

**Table 3:** Main adverse events of any cause in 12 months

Variables	Standard (n = 23)		Sequential (n = 30)		*P* value
	All Grade	Grade 3/4	All Grade	Grade 3/4	
Total	22 (96)	4 (17)	16 (53)	0 (0)	0.010/0.008
Mouth ulceration	22 (96)	2 (9)	12 (40)	0 (0)	
Hypercholesterolemia	14 (61)	1 (4)	6 (20)	0 (0)	
Hypertriglyceridemia	11 (48)	1 (4)	7 (23)	0 (0)	
Menstrual disorder	8 (44)	2 (11)	3 (10)	0 (0)	
Gastritis	7 (30)	0 (0)	2 (7)	0 (0)	
Urinary tract infection	7 (30)	1 (4)	3 (10)	0 (0)	
Headache	5 (22)	0 (0)	2 (7)	0 (0)	
Non-infectious pneumonia	5 (22)	0 (0)	0 (0)	0 (0)	
Fatigue	5 (22)	1 (4)	1 (3)	0 (0)	
Liver dysfunction	5 (22)	0 (0)	2 (7)	0 (0)	
Renal dysfunction	5 (22)	0 (0)	2 (7)	0 (0)	
Rash	4 (17)	0 (0)	2 (7)	0 (0)	
Anemia	4 (17)	0 (0)	2 (7)	0 (0)	

## Discussion

In recent years, many studies focused on the pathogenesis of TSC-related renal AML. It was found that TSC gene inactivation leads to the inactivation of inhibitory protein in the upstream of mTOR signaling pathway, abnormal activation of mTOR protein, excessive cell proliferation and large amount of angiogenesis, hence, AML is formed in the kidney [6]. Hence, mTOR inhibitors represented by rapamycin and everolimus can directly inhibit mTOR activity and stop the growth or reduce the volume of renal angiomyolipoma [7, 8]. In a multicenter randomized controlled prospective clinical study (EXIST-2), 79 patients clinically diagnosed with TSC-associated AML were treated with everolimus for 6 months, 42% of patients had a more than 50% volume reduction of renal AML lesions, compared to 0 in the control group [3]. In this study, everolimus had an acceptable safety profile, adverse reactions were tolerable, and the incidence of stomatitis and acne-like lesions was higher than that of the control group, while only 4% of patients terminated treatment due to adverse reactions. Ninety percent of the patients enrolled in this study were white, data on the Asian population was lacking.

Based on the previous experience of molecular targeted drugs in the treatment of advanced renal cancer, Chinese patients have poor tolerance to molecular targeted drugs, and most of them need to be adjusted for dosage. In the clinical application of everolimus in the Asian population, studies found that the Asian population had a higher incidence of adverse reactions [9]. Therefore, more and more researchers began to explore whether the previous everolimus 10 mg QD continuous administration regimen was suitable for the Asian population. In a non-prospective open study in a Chinese population, 18 TSC-RAML patients received everolimus 10 mg QD for 12 months, 67% of them had tumor volume reduction by more than 50%, and the incidence of adverse reactions was 100%, which was significantly higher than the results of EXIST-2 [9]. Taiwan scholars tried to treat 11 TSC-RAML patients with low-dose everolimus, starting at 2.5 mg QD, adding to 5 mg QD according to patient tolerance, 5 patients maintained 2.5 mg treatment, tumor volume decreased by 10.6–65.2%, and 3 patients experienced progress during follow-up. In 6 patients, tumor volume was reduced by 42.5–70.6% after incrementing to 5 mg, and no progress was observed during follow-up [10]. In another study from Japan, intermittent therapy was applied in 26 patients with TSC-RAML, the initial dose of everolimus was 10 mg QD for continuous administration. If the tumor diameter was less than 4 cm or the tumor volume did not change, the drug was discontinued. If the tumor volume reached 70% of that before treatment, the drug was restarted. The initial tumor volume decreased by an average of 67%, and 18 patients developed tumor progression and were readministered after discontinuation of the drug for 3–9 months (average of 6 months) [11]. Despite a significant reduction in the incidence of adverse events and medical expenses, both low-dose and intermittent treatment were less effective in tumor control than the standard regimen of 10 mg continuous administration and were therefore not optimal treatment options.

In our preliminary clinical work, we found that the blood drug concentration of the Chinese patients after treatment with everolimus was significantly higher than that of EXIST-2, which was similar to molecular targeted drugs in the treatment of advanced renal cell carcinoma. Hence, we drew on the experience of dose reduction therapy [12, 13]. According to their willingness and economic conditions, some patients were treated with dose-titration sequential therapy, and the initial dose of everolimus 10 mg QD was given for 3 months. After that, the dose was adjusted to 5 mg QD. Compared with standard treatment, sequential treatment showed no significant difference in tumor control and skin lesion control rate, while the incidence and severity of adverse reactions were significantly reduced, and the rate of drug reduction or interruption due to adverse reactions was also significantly lower than that of the standard treatment group. At the same time, the direct cost of patients in the sequential treatment group was significantly lower than those in the standard treatment group. More recently, a Japanese cohort has assessed the safety and efficacy of low-dose everolimus treatment in TSC patients with renal dysfunction or low body weight. Though the average blood everolimus trough level was lower (7.7 ± 3.1 vs. 12.2 ± 5.7 ng/mL), low-dose everolimus treatment (5 mg QD) can obtain similar disease control and lower incidence of adverse effects compared to conventional-dose group [14]. These results partly supported our findings.

In the present study, 35 (66%) patients had an AML with the longest diameter of at least 8 cm. However, in EXIST-2, less than one-third of all patients had AML to this extent of size. This significant difference may be due to different disease characteristics of the population. Two Chinese cohorts have described the similar results. Cai et al. reported 14/18 (77.8%) patients had an AML with the longest diameter of at least 8 cm [9]. Ni et al. reported 38/82 (46.3%) patients had an AML with the longest diameter of at least 10 cm [15]. The median time to tumor response in this study was 1.2 months, compared with 2.9 months in the EXIST-2 study. This may be related to the selected observation time, but this result suggests that most patients can achieve good clinical outcomes within 1 month after the administration of everolimus. In our group, the response rate of skin lesions after 1 year was 39%, which was significantly better than 23% in the study of EXIST-2. The incidence of overall and grade 3/4 adverse reactions at 3 months were higher than the results of the EXIST-2 study, especially the incidence of oral mucositis. However, after 12 months of sequential treatment, the incidence of adverse reactions was significantly lower than the data of EXIST-2, and no grade 3/4 adverse reactions occurred. Major adverse events included oral mucositis, hypertriglyceridemia, hypercholesterolemia, menstrual disorders, urinary tract infection, rash, headache, gastritis, etc., which generally did not require treatment or were relieved by temporary reduction or withdrawal of medication [16, 17]. Common grade 3/4 adverse reactions included oral mucositis, hypertriglyceridemia, hypercholesterolemia and abnormal liver function. There were 3 patients with intermittent epilepsy who had seizures at the beginning of medication, which should be alerted and generally relieved after 1–2 months. Adult TSC patients who still have seizures should be combined with antiepileptic drugs at the beginning of treatment, and be closely monitored for seizures. In the study of EXIST-2, the average blood trough concentration of everolimus was 7.63 ng/ml at 2 weeks and 9.37 ng/ml at 24 weeks. In this study, the mean serum trough concentrations of the two groups at 3 and 6 months were 19.63 ng/ml versus 20.09 ng/ml and 15.45 ng/ml versus 9.07 ng/ml, respectively. The mean serum trough concentration in 10 mg QD patients was 19.83 (8.03–54.5) ng/ml, while that in 5 mg QD patients was 10.9 (1.2–29.5) ng/ml. Previous studies suggested that the blood concentration of everolimus should be maintained between 5 and 15 ng/ml, but some patients were still effective at 3 ng/ml. Therefore, some scholars believed that as long as the ideal effect was obtained, the individualized drug administration scheme was feasible [18]. The results of serum concentrations in this study further supported the feasibility of sequential therapy in Chinese patients.

There are several limitations that need to note. First of all, the present study is a prospective cohort study. Due to the study design, the patients were not randomly assigned to two groups. It was difficult to control the grouping factors. The potential confounding factors and selection bias may affect the results. Second, patients were treated in our single center, and the sample size was relatively small. Further studies with large patients will provide more sufficient basis for the rationality of the scheme.

In summary, the mTOR inhibitor represented by everolimus has become the preferred treatment for patients with TSC associated AML at a stable stage. Sequential therapy has brought equal clinical benefit and good safety to Chinese patients with TSC-AML, and effectively save medical expenses.

## Data Availability

All data generated or analyzed during this study are included in this published article.
